# The global *Microcystis* interactome

**DOI:** 10.1002/lno.11361

**Published:** 2019-11-19

**Authors:** Katherine V. Cook, Chuang Li, Haiyuan Cai, Lee R. Krumholz, K. David Hambright, Hans W. Paerl, Morgan M. Steffen, Alan E. Wilson, Michele A. Burford, Hans‐Peter Grossart, David P. Hamilton, Helong Jiang, Assaf Sukenik, Delphine Latour, Elisabeth I. Meyer, Judit Padisák, Boqiang Qin, Richard M. Zamor, Guangwei Zhu

**Affiliations:** ^1^ Plankton Ecology and Limnology Laboratory, Department of Biology The University of Oklahoma Norman Oklahoma; ^2^ Program in Ecology and Evolutionary Biology and the Geographical Ecology Group, Department of Biology The University of Oklahoma Norman Oklahoma; ^3^ Department of Microbiology and Plant Biology and Institute for Energy and the Environment The University of Oklahoma Norman Oklahoma; ^4^ Institute of Marine Sciences, The University of North Carolina at Chapel Hill Morehead City North Carolina; ^5^ Department of Biology James Madison University Harrisonburg Virginia; ^6^ School of Fisheries, Aquaculture, and Aquatic Sciences Auburn University Auburn Alabama; ^7^ Australian Rivers Institute and School of Environment and Science, Griffith University Nathan Queensland Australia; ^8^ Department of Experimental Limnology, Leibniz Institute for Freshwater Ecology and Inland Fisheries, Stechlin, and Institute for Biochemistry and Biology Potsdam University Potsdam Germany; ^9^ Environmental Research Institute, University of Waikato Waikato New Zealand; ^10^ State Key Laboratory of Lake Science and Environment, Nanjing Institute of Geography and Limnology Chinese Academy of Sciences Nanjing China; ^11^ Israel Oceanographic and Limnological Research The Yigal Allon Kinneret Limnological Laboratory Migdal Israel; ^12^ Université Clermont Auvergne CNRS, LMGE Aubière Cedex France; ^13^ Institute for Evolution and Biodiversity, University of Münster Münster Germany; ^14^ Department of Limnology Institute of Environmental Science, University of Pannonia Veszprém Hungary; ^15^ Grand River Dam Authority Vinita Oklahoma

## Abstract

Bacteria play key roles in the function and diversity of aquatic systems, but aside from study of specific bloom systems, little is known about the diversity or biogeography of bacteria associated with harmful cyanobacterial blooms (cyanoHABs). CyanoHAB species are known to shape bacterial community composition and to rely on functions provided by the associated bacteria, leading to the hypothesized cyanoHAB interactome, a coevolved community of synergistic and interacting bacteria species, each necessary for the success of the others. Here, we surveyed the microbiome associated with *Microcystis aeruginosa* during blooms in 12 lakes spanning four continents as an initial test of the hypothesized *Microcystis* interactome. We predicted that microbiome composition and functional potential would be similar across blooms globally. Our results, as revealed by 16S rRNA sequence similarity, indicate that *M. aeruginosa* is cosmopolitan in lakes across a 280° longitudinal and 90° latitudinal gradient. The microbiome communities were represented by a wide range of operational taxonomic units and relative abundances. Highly abundant taxa were more related and shared across most sites and did not vary with geographic distance, thus, like *Microcystis*, revealing no evidence for dispersal limitation. High phylogenetic relatedness, both within and across lakes, indicates that microbiome bacteria with similar functional potential were associated with all blooms. While *Microcystis* and the microbiome bacteria shared many genes, whole‐community metagenomic analysis revealed a suite of biochemical pathways that could be considered complementary. Our results demonstrate a high degree of similarity across global *Microcystis* blooms, thereby providing initial support for the hypothesized *Microcystis* interactome.

Seasonally recurrent harmful algal blooms, particularly those of toxic cyanobacteria (cyanoHABs), are a global phenomenon of growing concern impacting water quality, ecosystem services, and human health associated with freshwater systems (Paerl and Otten 2013). Accelerating eutrophication and climate change (e.g., rising temperatures and shifting hydrological regimes) has resulted in the proliferation, intensification, and prolongation of cyanoHABs around the world (Carey et al. 2012; O'Neil et al. 2012; Paerl and Paul 2012; Mantzouki et al. 2018). Water quality in freshwater systems is intimately linked to anthropogenic activities. With rapidly expanding agricultural and urban development, as well as prolonged stratification periods due to global warming, many systems have become eutrophic or at risk of eutrophication. Although there is debate regarding the roles of specific nutrients in cyanoHAB dynamics (Conley et al. 2009; Paerl et al. 2011, 2016; Schindler 2012; Schindler et al. 2016), there is general agreement that increased nutrient inputs lead to increases in cyanobacterial biomass. CyanoHABs can alter ecosystem function by causing anoxia, depleting dissolved nutrients, and shifting zooplankton communities, all which alter carbon flows (Paerl and Otten 2013). Such blooms can produce hundreds of secondary metabolites, including hepatotoxins, neurotoxins, and dermatotoxic irritants, all of which can pose serious health threats to humans, livestock, and wildlife (Carmichael 2001; Huisman et al. 2018).

CyanoHABs, once thought to be homogeneous populations, are now known to be accompanied by a diverse suite of heterotrophic bacteria (Eiler and Bertilsson 2004; Steffen et al. 2012; Xu et al. 2018), which may play an important role in cyanobacterial bloom health and duration. First, Bell and Mitchell (1972) defined this potential interactive relationship between cyanobacteria and heterotrophic bacteria as the “phycosphere” (Paerl and Kellar 1978, 1979; Paerl and Millie 1996). It is well known that cyanobacteria generate abundant dissolved organic carbon resources to the benefit of nearby heterotrophs that can subsequently return benefits to the cyanobacteria, including removal of reactive oxygen species, CO_2_ generation, and nutrient recycling (Dziallas and Grossart 2011, 2012; Steffen et al. 2012; Paerl and Otten 2013). Moreover, *Microcystis* has been shown to alter ambient environmental conditions by decreasing oxygen concentrations and light availability (Paerl and Otten 2016), as well as by altering CO_2_ and pH levels (Havens 2008), which are likely to affect nearby bacteria. Indeed, studies have found that cyanobacterial bloom species strongly impact bacterial community composition (e.g., *Nodularia*—Salomon et al. 2003, *Microcystis*—Li et al. 2011; Steiner et al. 2017). Specifically, heterotrophic bacteria can form close‐knit aggregates with *Microcystis*, and studies have shown greater similarity between attached Bacteria and Archaea communities than between free‐living assemblages during *Microcystis* blooms (Cai et al. 2014; Yang et al. 2017; Batista et al. 2018; Xu et al. 2018).

One reason for this close association could be that, like other Bacteria and Archaea (Swan et al. 2013; Giovannoni et al. 2014), Cyanobacteria have small genomes compared with eukaryotes (Herdman et al. 1979; Humbert et al. 2013). While this may be beneficial for rapid reproduction and evolution, it is not necessarily conducive for cyanoHAB formation. Genome reduction can lead to loss of functions (Giovannoni et al. 2014), but can also confer a selective advantage if the organism can obtain the lost function through a public good as described by Morris et al. (2012) in the Black Queen Hypothesis. This hypothesis suggests that natural selection can act on “leaky” functions where a public good is produced and available to the whole community. Coupled with selection toward smaller genomes to reduce replication‐related fitness costs, some members of the community can receive metabolic products as public goods, which are useful metabolites or other necessary resources that are leaked into the cell‐external environment. With such products available extracellularly, these “leaky” functions become dispensable and once lost confer a selective advantage to that organism (Pande and Kost 2017). Garcia et al. (2015) proposed that this coevolved community of synergistic and interacting bacteria species was a bacterial community microbiome or “interactome,” analogous to the microbiome concept described for humans (Human Microbiome Project Consortium 2012), soils (Fierer 2017), and coral reefs (Bourne et al. 2013). We hypothesize that the bacteria associated with *Microcystis* may be providing functions that help sustain it during blooms.

Given the small size of bacterial genomes and presumed ubiquity of *Microcystis aeruginosa* (Kützing) Kützing, if there exists a mutualistic interactome, we would predict a microbiome of a certain species of associated bacteria or perhaps metabolic functions to be preserved across geographically distinct *M. aeruginosa* blooms. We would also expect communities to be more similar than predicted by traditional biogeographic theory, where community similarity is expected to decrease with increasing geographic distance (MacArthur 1984; Nekola and White 1999; Green and Bohannan 2006; Nemergut et al. 2013). As a first test of this prediction, we examined community composition and function of the *M. aeruginosa* bloom microbiomes from 12 lakes across four continents, addressing the specific prediction that if global *Microcystis* blooms were composed of the same taxon, the blooms would support and require a similar suite of bacterial‐provided functions, thus leading to highly similar bacterial communities across *Microcystis* blooms regardless of geographic location.

## *Methods*

### Sample collection

**Figure 1:**
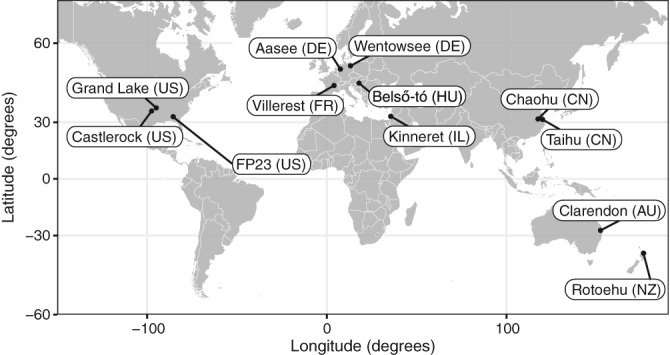
Location of the 12 lakes across the globe. These samples represent a 280° longitudinal and 90° latitudinal gradient.

Samples were collected from 12 lakes spanning the globe during the peak of the *Microcystis* blooms between May 2016 and July 2017 (Fig. 1). Three surface water samples were taken from each lake with a clean 1000‐mL beaker or Erlenmeyer flask and set aside undisturbed for 10 min to allow the cyanobacteria to float to the surface. Concentrated *Microcystis* biomass was poured off the top of the flask or beaker through a Nitex screen (100 μm pore size) stretched between a PVC pipe and PVC coupler to collect large *Microcystis* aggregates on the screen. Each Nitex screen was rolled up using forceps and transferred into 2‐mL screw cap tubes containing 1.0 mL DNA preservative (DNA/RNA Shield, Zymo Research). This process was carried out for each water sample. Tubes were stored at −20°C until shipping. They were shipped at ambient temperature and once received were stored at −20°C prior to extraction.

### DNA extraction and sequencing

DNA extraction from the preserved samples was performed using Zymo Research *Quick*‐DNA Fecal/Soil Microbe Miniprep Kits (Zymo Research) following the manufacturer's recommended protocol. The procedure involved placing the Nitex screen into the lysis tube with glass beads, followed by mixing with a Bead Beater vortex mixer (BioSpec products) for 2 min as a first step in the extraction process. Extracted DNA was collected by decanting and used as a template for amplifying bacterial 16S rRNA gene through PCR. Forward (S‐D‐Bact‐0341‐b‐S‐17, 5′‐CCTACGGGNGGCWGCAG‐3′) and reverse primers (S‐D‐Bact‐0785‐a‐A‐21, 5′‐GACTACHVGGGTATCTAATCC‐3′) were used for targeting the V3 and V4 regions of bacterial 16S rRNA gene (Klindworth et al. 2013). Each 50‐μL PCR reaction mix contained 2 μL of template (~ 30 ng), 2 μL of each primer (0.4 μM, final concentration), and 25 μL PCR master mix containing DreamTaq polymerase (Thermo Fisher Scientific). Thermocycler conditions were as follows: 94°C for 3 min followed by 28 cycles of 94°C for 30 s, 55°C for 60 s, and 72°C for 75 s, and a final elongation step at 72°C for 10 min. PCR products were visualized on 1% agarose gels to confirm amplification and then purified by QIAquick PCR purification kit (Qiagen) to remove primers. From each purified sample, 4 μL were added to a second PCR mixture containing barcoded primers for multiplexed Illumina sequencing. Through re‐amplification for another eight cycles in the second PCR reaction, each sample received a unique “barcode” sequence as previously described (Wawrik et al. 2012). The secondary PCR products were quantified with the Qubit dsDNA BR Assay kit (Life Technologies) on a Qubit 2.0 Fluorometer. Amplicons of all samples were pooled in an equimolar amount. Pooled samples were purified using a QIAquick PCR Purification Kit (Qiagen) and requantified with the Qubit. Paired‐end sequencing of the library was performed at the Oklahoma Medical Research Foundation, using the MiSeq Reagent Kit (v3) with the read length set to 2 × 300 base pairs (bp).

Shotgun metagenomics was used to profile functional potential and to recover whole genome sequences from the *Microcystis* and microbiome communities. Metagenomic sequencing was performed on two replicates per sample. A 300‐bp paired‐end library was constructed according to the instructions from Illumina. The libraries were sequenced on an Illumina Genome Analyzer IIx at the Oklahoma Medical Research Foundation. Eighteen metagenomes (two per lake) were multiplexed on two lanes, and a median total of ~ 48 million raw paired‐end reads was obtained for each sample (range: ~ 32–96 million, due to variations in library loading).

### Sequence processing and analysis

The 16S raw sequence data was processed via the QIIME pipeline (V1.9.1) (Caporaso et al. 2010), integrated with UPARSE‐OTU algorithm. Paired‐end reads were joined using the join_paired_ends.py function according to the SeqPrep method (https://github.com/jstjohn/SeqPrep) with a minimum overlap of 150 bp. The quality‐joined fragments were demultiplexed and primer sequences removed. After using the FASTQ Quality Filter (*q* = 20, *p* = 80) to remove unqualified sequences, the remaining fragments were clustered into operational taxonomic units (OTUs) at the 97% similarity level with UPARSE OTU clustering (Edgar 2013) which generates a representative set of high‐quality OTU sequences and filters out chimeric sequences via the de novo mode (using usearch 11.0.667; Edgar 2010). Taxonomic annotations were assigned to each high‐quality OTU sequence by RDP's naive Bayesian rRNA Classifier (Wang et al. 2007) against the SILVA SSUv132 Reference Database (Quast et al. 2012), at the confidence threshold of 80%. To perform phylogenetic analysis, OTU sequences were also aligned against the SILVA database with PyNAST (Caporaso et al. 2009), filtered, and a phylogenetic tree constructed with FastTree (Price et al. 2010). OTUs that failed in alignment or were classified as either eukaryote, archaea, chloroplast, or mitochondria were discarded from the OTU table, as were OTUs with fewer than 100 counts summed across samples.

To compare the community structure of the *Microcystis* microbiome among lakes, we extracted non‐cyanobacterial OTUs from the OTU table, and then retrieved their associated representative sequences. A phylogenetic tree with non‐cyanobacterial OTU sequences was constructed, as detailed above. OTU tables without cyanobacteria were rarified to the number of reads of the sample with the fewest reads. The rarified OTU table with pooled replicates was used for all downstream diversity calculation and statistical analysis.

Following quality trimming (Trimmomatic v 0.39; Bolger et al. 2014), with reads shorter than 20 bp being discarded and removal of human genes (MetaWRAP; Uritskiy et al. 2018), the clean metagenomic data were assembled into contigs by de novo assembly of each sample sequence using metaSPAdes (SPAdes v.3.13.0; Bankevich et al. 2012). The interactome (i.e., *Microcystis* and its microbiome) metagenomic assembled genomes (MAGs) were generated for each lake using three tools with default options: MaxBin (v.2.2.6) (Wu et al. 2015), MetaBAT (v. 2.12.1) (Kang et al. 2015), and CONCOCT (v. 1.0.0) (Alneberg et al. unpubl. preprint doi: arXiv:1312.4038v1 [q‐bio.GN]), followed by integration using DAS Tool (using a threshold of ≥ 70% genome completeness) (v. 1.1.1) (Sieber et al. 2018). The complete MAGs were then divided into two groups, *Microcystis* and heterotrophic bacteria, and pooled across the lakes. Protein‐encoding genes of each group were annotated from the contigs with Prokka (v1.13.3) (Seemann 2014). Duplicate genes were removed with CD‐HIT‐EST (v 4.6) (Hahn et al. 2016). Genes were then converted into protein sequences using Prokka. The protein sequences of each group were annotated to Kyoto Encyclopedia of Genes and Genomes (KEGG) orthologies to characterize individual gene functions using GhostKOALA (Kanehisa et al. 2016). To calculate the gene abundance in each sample, all KEGG annotated genes were first aligned with the clean reads by bowtie2 alignment software (Langmead and Salzberg 2012). The number of reads mapping to each gene was extracted using the SAMtools (v1.3.1) “idxstats” command. The abundance of each gene in all samples was calculated by get_count_table.py (https://github.com/edamame-course/Metagenome/blob/master/get_count_table.py). To compare the functions contributed by the microbiome to those of the *Microcystis*, we constructed complete KEGG pathways (no less than one missing gene). Due to the high phylogenetic and functional similarities found across lakes (see the Results section), we pooled *Microcystis* MAGs and microbiome MAGs (Li et al. 2018), respectively, in order to generate the pathways. We uploaded the KO numbers into the online KEGG mapper to construct the complete KEGG pathways.

Due to the low depth of coverage for *Microcystis* microbiome genes, we attempted to maximize the probability of identifying functional potential in the *Microcystis* microbiomes by repeating the process outlined above for MAGs using the total metagenomic data. We separated the *Microcystis* reads from the microbiome reads by aligning the clean metagenomic data with 16 *Microcystis* genomes (10 *M. aeruginosa*, 1 *Microcystis viridis*, 1 *Microcystis panniformis*, 2 *Microcystis flos‐aquae*, and 2 *Microcystis wesenbergii*) using bwa v0.7.15 (Li and Durbin 2009) and splitting the data based on this alignment. The reads were then assembled into contigs (SPAdes v3.1.1; Bankevich et al. 2012) and the contigs for each lake (*Microcystis* and the microbiome kept separate) were pooled across lakes into a single group and annotated (Prokka v1.13.3; Seemann 2014). Duplicate genes were then removed with CD‐HIT‐EST (v 4.6) (Hahn et al. 2016). The protein sequences of each group were annotated to KEGG orthologies, the gene abundance mapped to each lake, and the data pooled and complete KEGG pathways constructed as described above.

Raw sequence data in this study have been deposited at the National Center for Biotechnology Information website under BioProject accession number PRJNA575023.

### Statistical analyses

All statistical analyses were completed in the R statistical environment v.3.5.1 (R Development Core Team 2018), except where noted otherwise. We first tested for associations between geographic distance and community dissimilarity across samples. We calculated the great circle geographic distance between sites using the “rdist.earth” function (fields v.9.6). We calculated Bray–Curtis dissimilarity using “vegdist” (vegan package v.2.5‐3) (Oksanen et al. 2013). UniFrac values, weighted by abundance, were generated in QIIME. Generalized linear models (GLM) were used to assess the relationship between geographic distance and community dissimilarity measures (using default family = Gaussian, link = identity). Deviance explained by GLMs coupled with *p*‐values was used to assess the significance and strength of the relationship.

To examine the phylogenetic relationship among *Microcystis* microbiome bacteria *within* each sample (α‐diversity), we calculated mean‐nearest‐taxon‐distance (MNTD) and the nearest‐taxon‐index (NTI) (Webb 2000) using the “mntd” and “ses.mntd” commands in the Picante package v.1.7 in R (Kembel et al. 2010; Swenson 2014). NTD is a measure of phylogenetic distance between each OTU within a sample and its closest relative in the same sample. The mean is then calculated across all phylogenetic distances in a sample to give a value of phylogenetic relatedness. To determine whether observed phylogenetic community composition was more or less related (or structured) than predicted by chance, null models were built by randomly shuffling the taxa within each community across the tips of the phylogeny (null.model = “taxa.labels” in “ses.mntd”) and recalculating MNTD 999 times (Stegen et al. 2012). The resulting NTI (which is the negative output of “ses.mntd”) distribution displays the number of standard deviations that the observed MNTD is from the mean of the null MNTD values. An αNTI value less than −2 indicates taxa within the community are more distantly related than by chance (phylogenetically overdispersed), a value greater than 2 indicates taxa are more closely related than expected by chance (phylogenetically clustered), and αNTI values between 2 standard deviations of 0 indicate that the observed community is no different from random (Webb 2000; Stegen et al. 2012). A two‐sample *t*‐test was used to assess whether the mean αNTI values from across all communities was significantly different from that of the null distribution.

We used beta‐mean‐nearest‐taxon‐distance (βMNTD) and beta‐nearest‐taxon‐index (βNTI) to quantify the phylogenetic distance among samples (*see* Swenson 2014 for R code). Similar to its α‐diversity analog, βMNTD is the mean of the phylogenetic distances between each OTU in a given sample to their nearest relative in the comparison sample. Null distributions were generated by randomizing OTUs across the phylogeny and recalculating βMNTD 999 times (Stegen et al. 2012). As before, βNTI is the number of standard deviations that the observed βMNTD is away from the mean of the null distribution. A value of βNTI less than −2 indicates more phylogenetic relatedness between samples than expected by chance, and a βNTI greater than 2 indicates less phylogenetic relatedness between the two communities than expected by chance (i.e., species in these communities are more phylogenetically distant from each other). A two‐sample *t*‐test was used to determine if the mean βNTI for two communities was significantly different from the null expectation.

The abundance of KEGG genes for the *Microcystis* microbiome for each lake (mapped from the pooled total metagenomic data) was used to measure the community functional dissimilarity using Bray–Curtis (pairwise comparisons between lakes). GLM were used to assess the relationship between geographic distance and community functional dissimilarity measures (using default family = Gaussian, link = identity). To explore important biogeochemical pathways that may be shared or different between the *Microcystis* and their associated bacteria, we compared the complete (no more than one gene missing) KEGG pathways between *Microcystis* and the microbiome. Due to the low depth of coverage of the microbiome, we used both the MAGs complete KEGG pathways and the total metagenome complete KEGG pathways for this analysis to maximize the probability of identifying a complete pathway.

## *Results*

The sampled lakes spanned a 280° longitudinal and 90° latitudinal gradient ranging from 444 to 11,777 km apart and represented *M. aeruginosa* blooms from four continents (Fig. 1). After removal of Eukaryota, singletons, chloroplasts, and mitochondrial reads, 3.25 million high‐quality reads were represented by 454 unique (97% similarity) bacterial OTUs. *Microcystis* (927 total OTUs) was dominant in 10 of the 12 lakes and ranged from 65% to 84% of total sequence abundance. Both Taihu and Wentowsee communities were made up of less than 50% *Microcystis* (48% and 5%, respectively) sequences suggesting the lakes were not at peak bloom phase during sampling. As such, these two lakes were removed from subsequent analyses. Castlerock was also removed from subsequent analyses due to a loss of one of the triplicate samples. All *Microcystis* OTUs with greater than 1000 total abundance were reidentified to species using NCBI BLAST function (Altschul et al. 1990) and were all identified as *M. aeruginosa*, accounting for 79–85% of the total *Microcystis* across lakes and 53–68% of all OTU abundances across lakes. This confirms that, at least at the level of 16S rRNA sequences, *M. aeruginosa* is a cosmopolitan bloom‐forming species.

**Figure 2:**
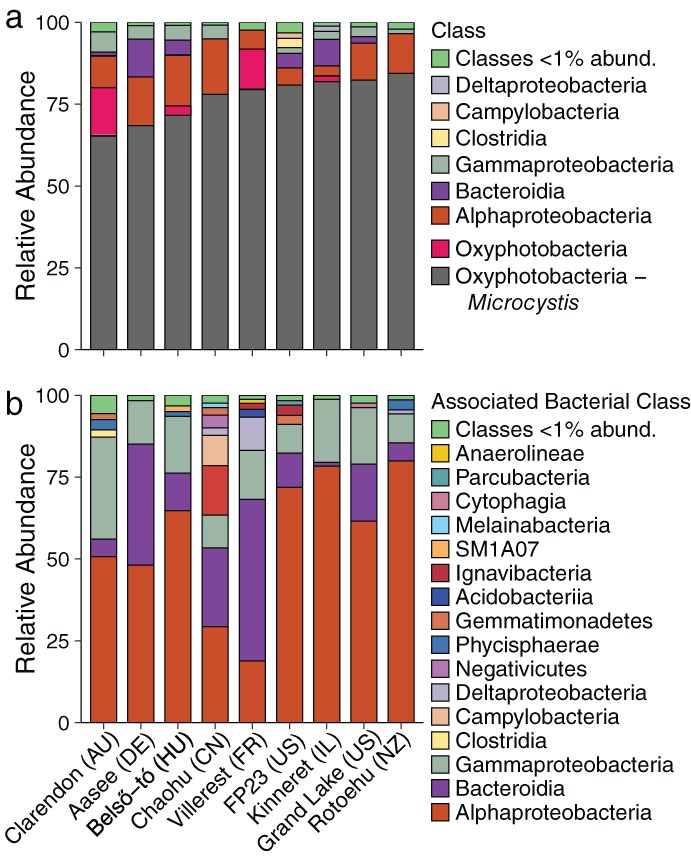
(**a**) Relative abundance of Bacteria classes or subclasses in the nine lakes. The lakes are arranged in order from left to right of increasing percent *Microcystis* in the community. Classes less than 1% of the total relative abundance were grouped together as a single group denoted “<1% abund.” Oxyphotobacteria (cyanobacteria) were split into two groups: *Microcystis* only in one and all other cyanobacteria in the second. (**b**) Relative abundance of non‐*Microcystis* (i.e., microbiome) bacterial classes.

**Figure 3:**
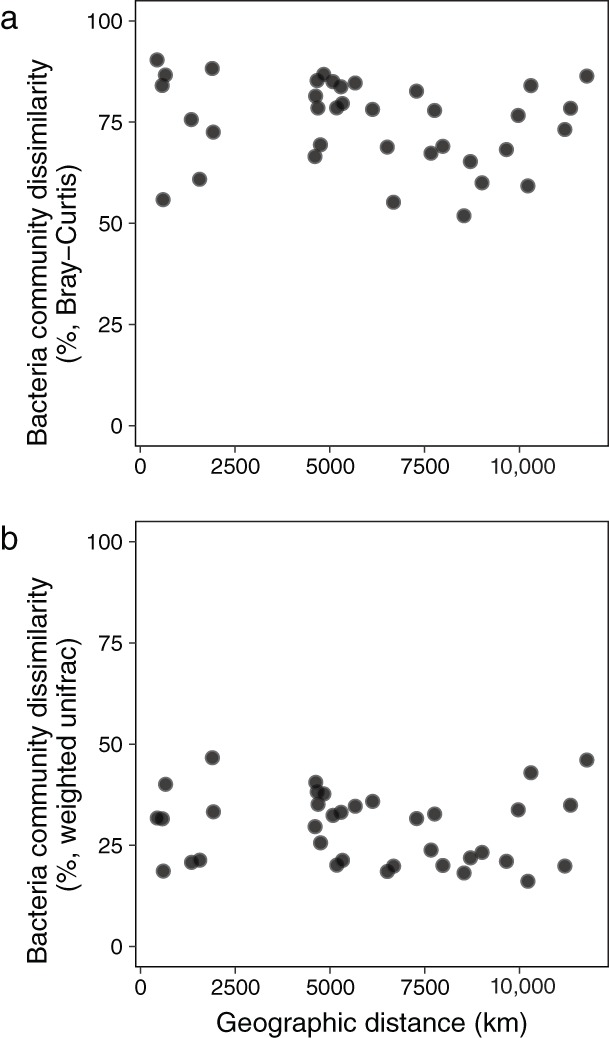
Scatter plots of community dissimilarity in the microbiome as related to geographic distance. (**a**) The nonsignificant (GLM deviance explained = 3.7%, *p* = 0.2) relationship between taxonomic Bray–Curtis dissimilarity and geographic distance where the higher Bray–Curtis values indicate fewer species in common between sites. (**b**) Abundance weighted UniFrac did not scale significantly by geographic distance (GLM DE = 0.82%, *p* = 0.55). Here, higher values of UniFrac indicate there is little overlap in species between communities whereas lower values indicate the communities are more similar.

The 454 non‐cyanobacterial OTUs were associated with 35 bacterial classes, with most OTUs identified as Alphaproteobacteria, Bacteroidia, Gammaproteobacteria, Clostridia, Campylobacteria, Deltaproteobacteria, Negativicutes, Phycisphaerae, Gemmatimonadetes, Acidobacteriia, Ignavibacteria, SM1A07, Melainabacteria, Cytophagia, Parcubacteria, and Anaerolineae (Fig. 2b). The abundance and taxonomy of the associated bacteria differed among sites (Fig. 2b) with Alphaproteobacteria, Bacteriodia, and Gammaproteobacteria classes contributing most sequences across sites. Bray–Curtis dissimilarity values were relatively high for all of the site comparisons indicating large differences in OTUs among sites (Fig. 3a). Weighted UniFrac, on the other hand, was generally low, suggesting that among sites, there were many shared taxa (or a few shared taxa with high abundances) with some degree of phylogenetic relatedness (Fig. 3b). These results together suggest that there was a wide range of relative abundances of taxa across all of the sites, including many low abundance rare taxa (contributing to high Bray–Curtis values), while the highly abundant taxa were more related and shared across most sites (contributing to lower UniFrac values). Neither of the community dissimilarity metrics were significantly correlated with geographic distance (Fig. 3a,b; BC GLM deviance explained = 4.1%, *p* = 0.2; UF GLM DE = 0.97%, *p* = 0.57).

**Figure 4:**
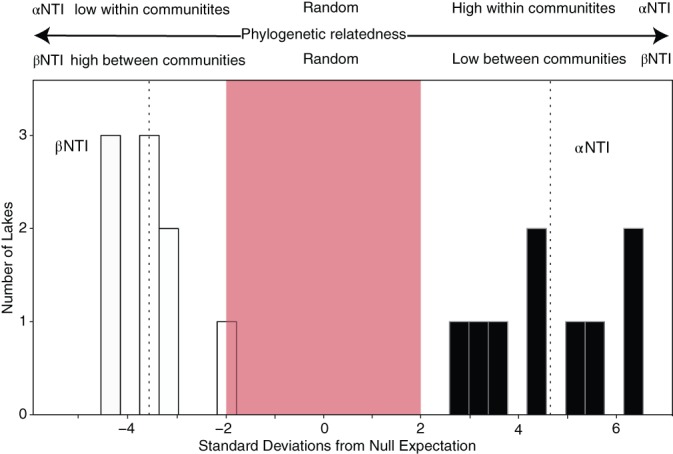
Distributions of *within* community phylogenetic relatedness (αNTI, nearest taxon index), and the phylogenetic relatedness *between* two communities (βNTI) of the nine sampled lakes. Values below −2 or above +2 SD from the null (indicated by the red rectangle) are statistically significantly different from random. Black dashed lines indicate the mean of the observed distributions. The mean of the αNTI distribution is 4.64 and the mean of the βNTI distribution is −3.58. αNTI is a measure of community phylogenetic structure and relatedness, where positive deviations from the null expectation indicate the species in the community are more phylogenetically related (clustered) than expected by chance (as seen here), and negative deviations indicate the species are more phylogenetically distant (overdispersed). The observed αNTI was significantly different from the null (*t* = 10.13, *p* < 0.001). βNTI measures phylogenetic relatedness between two communities with values greater than the null meaning lower relatedness than expected by chance and values lower than the null meaning higher relatedness than expected by chance (as seen here). Our βNTI is significantly different from random (*t* = −12.65, *p* < 0.001).

Phylogenetic α‐diversity analysis using mean nearest taxon distance and null model generation revealed on average that within lakes, bacteria in the *Microcystis* microbiome were more phylogenetically related than predicted by chance (Fig. 4, +αNTI; *t* = 10.13, *p* < 0.001). Among lakes, the *Microcystis* microbiome communities also were significantly more phylogenetically related than expected by chance (Fig. 4, βNTI; *t* = −12.65, *p* < 0.001).

A total of 866 million metagenomic sequencing reads were generated from the nine lake *Microcystis*‐microbiome communities. After trimming and filtering, 612 million (52.4–137.6 million per lake) clean reads were generated (Supplementary Table S1). Most of the clean metagenomic reads (55.2–76.6% per lake) belonged to *Microcystis*.

**Figure 5:**
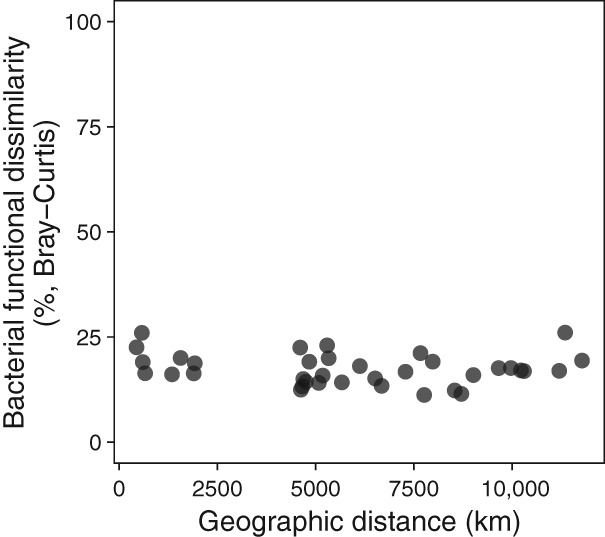
The dissimilarity between the *Microcystis* microbiome community's metagenomic function was not significantly correlated with geographic distance (GLM DE = 1.97%, *p* = 0.41) and was overall low (low values of Bray–Curtis dissimilarity).

Analysis of only whole genome data revealed nine *Microcystis* genome bins (one MAG per lake) and but only 43 microbiome bacterial genome bins (3–10 MAGs per lake), indicating extremely low depth coverage for the microbiome bacteria. After removing the duplicate genes and RNA genes, 156,445 and 39,880 protein‐coding genes were obtained from the microbiome and *Microcystis* genomes, respectively. Our analysis showed that 66,861 (∼ 42%) of the microbiome genes, and 14,188 (~ 35%) of *Microcystis* genes were successfully assigned to the KEGG orthology. By contrast, after removing duplicate and rRNA genes, analysis of the total metagenomic data produced 407,658 and 54,312 protein‐coding genes for the microbiome and *Microcystis*, respectively, of which 139,384 (more than twice the number compared with MAGs) and 17,817 (3629 more), respectively, were successfully mapped to the KEGG orthology. Microbiome gene diversity was similar across lakes (Fig. 5; low Bray–Curtis values) and did not differ with geographic distance between the lakes (Fig. 5; BC GLM DE = 1.97%, *p* = 0.41).

**Figure 6:**
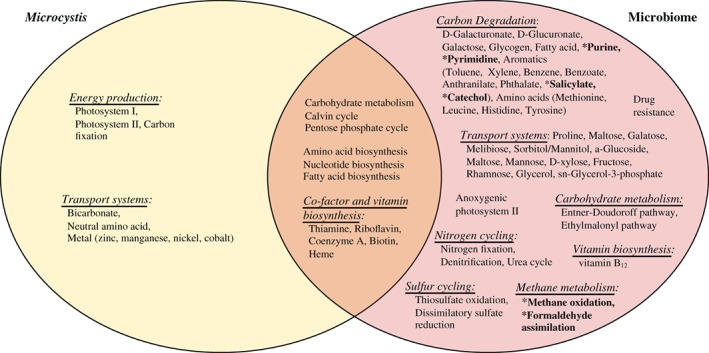
Venn diagram showing the distribution of complete or nearly complete (no more than one gene missing) KEGG modules in *Microcystis* and the microbiome bacteria. See Supplementary Table S2 for details and indication for involvement in major elemental cycling. KEGG modules in bold print with asterisks were detected in the full metagenome data but not in the *Microcystis* or the microbiome bacterial MAGs.

Shared pathways were mostly assigned to carbohydrate metabolism; amino acid, nucleotide, and fatty acid biosynthesis; and cofactor and vitamin biosynthesis (Fig. 6). The microbiome bacteria contained unique pathways for organic carbon transportation and degradation and vitamin B_12_ synthesis not found in *Microcystis*. Numerous pathways for organic carbon degradation (d‐galacturonate, D‐glucuronate, galactose, glycogen, fatty acid, purine, pyrimidine) and transportation (e.g., proline, maltose, galactose, maltose, mannose, D‐xylose, fructose, rhamnose, glycerol), as well as complete pathways for degradation of aromatics (mostly anthropogenic pollutants, such as toluene, xylene, benzene, phthalate) were also identified in the microbiome. Numerous important anaerobic bacterial pathways, including nitrogen fixation, denitrification, and dissimilatory sulfate reduction were also detected in the *Microcystis* microbiome, suggesting the anaerobic bacteria play an important role in nutrient cycling during *Microcystis* blooms in these lakes. With the exception of some genes related to carbon cycling (purine, pyrimidine, salicylate, and catechol degradation), as well as bacterial pathways involved in methane metabolism (methane oxidation and formaldehyde assimilation), the majority of potential biochemical function appeared to be associated with the 43 microbiome MAGs (Fig. 6 and Table S2).

## *Discussion*

Genome reduction leads to a loss of function (Giovannoni et al. 2014), necessitating interactions with community members capable of carrying out those functions (Morris et al. 2012; Garcia et al. 2015). With small genomes compared to other algae (e.g., 4.2 vs. 34.5 Mbp, Armbrust 2004; Gregory et al. 2007), *Microcystis* spp. are potentially missing some key metabolic functions (Steffen et al. 2012) and might be reliant on community members to fill in the metabolic gaps. Similar potential mutualist interactions have recently been mapped between *Microcystis* and their microbiome of associated bacteria (Xie et al. 2016; Li et al. 2018), corroborating the original idea of a “phycosphere” of functional interaction within the *Microcystis* aggregates (Bell and Mitchell 1972; Paerl and Kellar 1978, 1979; Paerl and Millie 1996). Moreover, bacteria have been shown to complement algae in marine ecosystems by excreting large amounts of exometabolites including growth factors and biosynthetic precursors, as well as processing toxic metabolites (Morris et al. 2011; Pérez et al. 2016; Lee et al. 2017; Wienhausen et al. 2017). Given the predominance of such data across aquatic system, we hypothesized *M. aeruginosa* blooms have a microbiome of certain species of associated bacteria or perhaps metabolic functions that will be preserved across geographically distinct *Microcystis* blooms.

*M. aeruginosa* was the most abundant *Microcystis* species across all lakes and this confirms that, at least at the level of 16S rRNA sequences, *M. aeruginosa* is a cosmopolitan bloom‐forming species. We found remarkable phylogenetic relatedness among associated bacteria, and similar function between sites, despite those bacteria being taxonomically distinct at the 16S rRNA level. We found no relationship between community composition dissimilarity and geographic distance (Fig. 3a, b), indicating no distance‐decay relationship as would be expected for dispersal‐limited species (Nekola and White 1999; Green and Bohannan 2006; Nemergut et al. 2013). We also conclude that the functional potential of microbial communities is more highly conserved than their taxonomic composition (Figs. 3a, 5). Similarly, Steffen et al. (2012) found that the cyanobacterial bloom‐associated bacterial communities across three lakes (Erie, Taihu, St. Marys) differed taxonomically, while being functionally similar. Thus, while OTU identity was variable across *Microcystis* microbiome bacteria, functional potential appears to have been quite similar providing evidence that under bloom conditions, *M. aeruginosa* is accompanied by a common suite of bacterial functionality, potentially forming an interactome.

We found that *Microcystis* microbiome bacteria have functional potential not found in *Microcystis*, and functional similarity was preserved globally. Pathways involving photosynthesis (excluding the anoxygenic photosystem II pathway) and carbon fixation were only detected in *Microcystis* (Fig. 6). The microbiome bacteria could potentially be contributing to carbon recycling within the aggregates as many of the pathways found only in the bacteria are related to carbohydrate breakdown (e.g., d‐galacturonate, galactose, and glycogen degradation) and transport (e.g., numerous transport systems). The microbiome bacteria are likely tightly associated with the carbon sources found in the *Microcystis* aggregates. Bacteria associated with phytoplankton blooms have been found to utilize the carbon source glycolate from phytoplankton (Lau et al. 2007; Paver and Kent 2010) and contain the glycolate oxidation pathway. Unfortunately, the KEGG database does not contain this gene or related pathways thus we were unable to detect it in our samples. Every metagenomic function database has its shortcomings, and for future studies we would recommend using multiple databases to cover as many genes and pathways as possible. For example, using the COG database (clusters of orthologous groups) to search for specific genes, we in fact found that the glcD gene, which is involved in the glycolate oxidation pathway, as well as the microcystin production genes (mcyA‐I) were present in the microbiome and the *Microcystis*, respectively, in all of our lakes. In addition, we found that the microbiome potentially contributes methane metabolism pathways (methane oxidation and formaldehyde assimilation) which are used to convert methane into a useable carbon form. Recently, cyanobacteria have been suggested to produce methane during blooms (M. Bižić et al. unpubl preprint, https://doi.org/10.1101/398958), although methane is typically produced by methanogenic archaea. While we did not analyze the archaea in our samples, other studies have found methanogenic archaea can be closely associated with cyanobacteria blooms (Batista et al. 2018), thus we would expect some methane production within aggregates.

In addition, the ethylmalonyl pathway was identified in the microbiome bacterial genomes. The ethylmalonyl pathway is a new acetate assimilation strategy in *Rhodobacter sphaeroides*, an anoxygenic phototrophic organism that lacks the key enzyme of the glyoxylate cycle, isocitrate lyase (Erb et al. 2007). Indeed, genes associated with anoxygenic photosystem II were identified in six microbiome MAGs (data not shown). Sulfur is a byproduct of anoxygenic photosynthesis and we also found evidence for thiosulfate oxidation and dissimilatory sulfate reduction pathways present in the microbiome bacteria. These results corroborate Li et al. (2018), who suggested that bacteria associated with *Microcystis* (termed epibionts by Li et al.) were essential for maintaining the redox balance and cycling different forms of sulfur within *Microcystis* aggregates. In addition, the vitamin B_12_ biosynthesis pathway, a necessary vitamin that *Microcystis* cannot produce, was only detected in the microbiome bacteria. Croft et al. (2005, 2006) proposed that most phytoplankton were likely auxotrophic for vitamin B_12_ and other essential vitamins. Indeed, previous studies have also suggested that vitamin B_12_ was provided to *Microcystis* by the associated bacteria and, moreover, that this relationship was mutually beneficial for both groups (Xie et al. 2016; Li et al. 2018).

Most *Microcystis*‐blooming lakes are typically recipients of urban and agricultural runoff. Correspondingly, we found the *Microcystis* microbiome contained the degradation pathways for many potentially harmful aromatic pollutants (e.g., benzene, benzoate, phthalate, etc.). *Microcystis* could be benefiting from pollutant degradation as many of these aromatics have been shown to inhibit phytoplankton growth (Häder and Gao 2015). Xie et al. (2016) also found a group of aggregate‐associated bacteria that contributed the whole benzoate degradation pathway to the community, again pointing toward mutualism between the *Microcystis* and the *Microcystis* microbiome. Together, these results support our hypothesis of a coevolved interactome. We also hypothesize that many of these microbiome functions (Fig. 6) are needed for growth and dominance by *M. aeruginosa* during bloom conditions.

Loss of necessary metabolic functions is more common in bacterial communities than previously thought (Hottes et al. 2013). Morris et al. (2012) found that the marine cyanobacterium, *Prochlorococcus*, has lost many oxidative‐stress genes and instead relies on other microbes for removal of hydrogen peroxide from the microenvironment. As such, *Prochlorococcus* may benefit from hydrogen peroxide removal via the actions of “helper” microbes in the form of a “leaky” public good (sensu Morris et al. 2012), and the smaller genome of *Prochlorococcus* affords it a selective advantage. A recent genomic study has shown that *M. aeruginosa* also has a reduced genome with remarkable redundancy consisting of a set of ~ 2400 core genes and a large, variable pangenome, an additional set of genes unique to different *M. aeruginosa* strains likely acquired through horizontal gene transfer (Humbert et al. 2013). We speculate that the variability within the *M. aeruginosa* pangenome could be due to differences in the need for specific functions across different environments. Horizontal gene transfer and the Black Queen Hypothesis provide two mechanisms for coping with changing environmental needs. “Leaky” functions may be lost from *Microcystis* when they are costly and a public good is available, as with the case of oxidative‐stress genes in *Prochlorococcus*. However, when that public good becomes less predictable, those functions may be recouped through horizontal gene transfer if the new conditions provide a selective advantage to carriers for regaining the function. Steffen et al. (2012) corroborated this hypothesis as they found microbiome functional potential remained static between two *Microcystis* blooms. However, in one of the lakes (Taihu), *Microcystis* was reliant on Proteobacteria for nitrogen assimilation and metabolism, while in the other lakes (Erie and St. Marys), *Microcystis* carried out those functions. In the present study, we also found that the microbiome bacteria were potentially the sole contributors of nitrogen fixation, denitrification, and the urea cycle pathways (Fig. 6). *Microcystis* cannot fix nitrogen so this may indicate that *Microcystis* is relying on public goods across multiple blooms. This interactome of *M. aeruginosa* and its microbiome could be an ideal system to test for “Black Queen” functions.

Alternatively, the similarities we see in the microbiome bacterial community could be due in part to the *Microcystis* bloom creating specific habitats that are selective of certain types of heterotrophic bacteria. As previously mentioned, *Microcystis* blooms can change local environments, creating large amounts of particulate organic matter that have been shown to be an important nutrient source for shaping the bacterial community (e.g., Fogg and Harold 1952; Bell and Mitchell 1972; Paerl and Gallucci 1985). We saw through βNTI analysis that *Microcystis* microbiome communities were significantly phylogenetically related (Fig. 4), with the negative value of βNTI indicating a common environmental filter across the lakes—*Microcystis* (sensu Dini‐Andreote et al. 2015). Similarly, multiple studies (Yang et al. 2017; Shi et al. 2018; Xu et al. 2018) have shown that the *Microcystis*‐associated (i.e., particle‐attached, which is equivalent to our use on *Microcystis* microbiome) bacterial community composition appeared to be heavily structured by the bloom compared to free‐living bacteria. Over the course of a *Microcystis* bloom, Parveen et al. (2013) found that *Microcystis* bloom aggregates provided habitat for a bacterial community distinct from the free‐living bacteria. In addition, specific bacteria have also been found utilize parts of bloom aggregates. For example, bacteria in the genus, *Sphingomonas*, actively break down toxins while associated with *Microcystis* blooms (Dziallas and Grossart 2011). This bloom‐as‐habitat hypothesis could also explain the differences in attached and free‐living bacteria described in previous studies (Yang et al. 2017; Shi et al. 2018; Xu et al. 2018). This hypothesis does not negate that *Microcystis* could be exchanging or receiving functions from these aggregate‐associated bacteria. Additional metagenomic and multi‐year bloom studies are needed to further parse these relationships.

The phylogenetic and functional similarities compared with the taxonomic dissimilarities we observed provide support to a growing body of evidence suggesting that community composition comparisons should be based on functional genes rather than strictly taxonomy (OTUs) (Burke et al. 2011; Oh et al. 2011). While clear similarities are shown using 16S rRNA genes, this method is taxonomically conservative and multiple different bacterial species could be represented by a single OTU. 16S rRNA genes represent a very small part of the genome, and evidence is growing which suggests that 16S rRNA is insufficient for distinguishing freshwater bacteria. For example, *Polynucleobacter* species, with 16S rRNA similarities ≥ 99%, have been shown to be distinct species based on whole genome comparisons and ecological isolation (Hahn et al. 2016). In *Microcystis*, a threshold of 98–99% similarity is suggested to be sufficient in distinguishing some species (Harke et al. 2016). In our study, all *Microcystis* OTUs with greater than 1000 sequence abundance were assigned to *M. aeruginosa* at the species level, thus we are confident in this taxonomy, but also recognize the need for more detailed taxonomic analysis to further investigate the diversity of *Microcystis* within a bloom beyond the 97% similarity threshold for 16S rRNA genes.

In the current study, we have used 16S rRNA taxonomy paired with metagenomic functional analysis to describe the community composition and potential function of the associated bacterial community in nine geographically distinct *Microcystis* blooms. The multiple samples across continents have allowed us to confirm that *M. aeruginosa* is a cosmopolitan bloom former. The phylogenetic and functional similarity of associated bacteria across sites and the sets of complementary pathways found between the *Microcystis* and associated bacteria support the presence of a synergistic interactome. These results highlight the need for deeper investigation into *Microcystis* taxonomic identity and both *Microcystis* and microbiome functional capabilities at different times before, during, and after a bloom to fully elucidate the interactome relationship between *M. aeruginosa* and its microbiome. We conclude that the coevolution within an interactome, that is, between *M. aeruginosa* and its microbiome, could help explain the global distribution of, and dominance by, *M. aeruginosa* in diverse freshwater ecosystems.

## Conflict of Interest

None declared.

## Supporting information


**Appendix**S1: Supporting Information 1
