# Supplementary material for: The global Microcystis interactome
Source: Limnol Oceanogr. 2019 Nov 19;65(Suppl 1):S194–207. doi: 10.1002/lno.11361 (PMC7003799; doi:10.1002/lno.11361)

**Supporting Information:**

The global *Microcystis* interactome

Cook, K.V.,[1,2] C. Li,[3], H. Cai[1], L.R. Krumholz,[3] K.D. Hambright,[1,2,*] H.W. Paerl,[4] M.M. Steffen,[5] A.E. Wilson,[6] M.A. Burford,[7] H.-P. Grossart,[8] D. P. Hamilton,[7,9] H. Jiang,[10] A. Sukenik,[11] D. Latour,[12] E.I. Meyer,[13] J. Padisák,[14] B. Qin,[10] R.M. Zamor,[15,a] and G. Zhu[10]

**Table S1.** Summary of metagenome data for each lake.

| Lake | category | contigs number | contigs bases(Mbp) | N50(bp) | Max length(bp) | protein-coding gene number |
|---|---|---|---|---|---|---|
| Belső-tó | Bacteria | 33496 | 27.1 | 765 | 74324 | 26699 |
| | *Microcystis* | 890 | 4.7 | 16388 | 81696 | 4267 |
| Chaohu | Bacteria | 23682 | 19.8 | 794 | 36145 | 18385 |
| | *Microcystis* | 5258 | 7.8 | 1944 | 54606 | 6268 |
| Clarendon | Bacteria | 82636 | 104.8 | 1392 | 321729 | 102844 |
| | *Microcystis* | 6740 | 10.9 | 2336 | 38246 | 9427 |
| FP23 | Bacteria | 155545 | 19.5 | 1454 | 49139 | 28976 |
| | *Microcystis* | 4816 | 7.9 | 2248 | 35883 | 6825 |
| Grand | Bacteria | 73551 | 92.3 | 1418 | 105344 | 89141 |
| | *Microcystis* | 9312 | 11.1 | 1418 | 57431 | 8881 |
| Kinneret | Bacteria | 26202 | 37.0 | 1817 | 101729 | 37307 |
| | *Microcystis* | 4943 | 9.1 | 2627 | 55640 | 8028 |
| Aasee | Bacteria | 36892 | 57.9 | 2441 | 299862 | 56457 |
| | *Microcystis* | 1122 | 5.5 | 19101 | 77987 | 5191 |
| Rotoehu | Bacteria | 26009 | 31.1 | 1368 | 41132 | 32313 |
| | *Microcystis* | 503 | 4.3 | 16710 | 49814 | 4098 |
| Villerest | Bacteria | 30454 | 40.5 | 1691 | 97662 | 36391 |
| | *Microcystis* | 2619 | 6.7 | 5702 | 54900 | 5927 |

**Table S2.** KEGG orthology numbers and functional pathways found in *Microcystis* and the microbiome bacteria, indicating involvement in C, N, P, or S cycling. Complete pathways are indicated by black fill ■, while modules with no more than one pathway missing, are indicated by blue fill [blue fill]. Empty cells indicate missing or partial (>1 missing) pathways.

| Pathway | *Microcystis* | Bacteria | Biogeo-chemical cycle |
|---|---|---|---|
| **Carbohydrate metabolism** | | | |
| M00001 Glycolysis (Embden-Meyerhof pathway), glucose => pyruvate | ■ | ■ | **C** |
| M00002 Glycolysis, core module involving three-carbon compounds | ■ | ■ | **C** |
| M00003 Gluconeogenesis, oxaloacetate => fructose-6P | ■ | ■ | **C, P** |
| M00307 Pyruvate oxidation, pyruvate => acetyl-CoA | ■ | ■ | **C** |
| M00009 Citrate cycle (TCA cycle, Krebs cycle) | [blue fill] | ■ | **C** |
| M00010 Citrate cycle, first carbon oxidation, oxaloacetate => 2-oxoglutarate | ■ | ■ | **C, S** |
| M00011 Citrate cycle, second carbon oxidation, 2-oxoglutarate => oxaloacetate | [blue fill] | ■ | **C** |
| M00004 Pentose phosphate pathway (Pentose phosphate cycle) | ■ | ■ | **C, P** |
| M00006 Pentose phosphate pathway, oxidative phase, glucose 6P => ribulose 5P | ■ | ■ | **C,P** |
| M00007 Pentose phosphate pathway, non-oxidative phase, fructose 6P => ribose 5P | ■ | ■ | **C,P** |
| M00580 Pentose phosphate pathway, archaea, fructose 6P => ribose 5P | [blue fill] | ■ | **C,P** |
| M00005 PRPP biosynthesis, ribose 5P => PRPP | ■ | ■ | **C,P** |
| M00008 Entner-Doudoroff pathway, glucose-6P => glyceraldehyde-3P + pyruvate | | ■ | **C,P** |
| M00308 Semi-phosphorylative Entner-Doudoroff pathway, gluconate => glycerate-3P | | ■ | **C,P** |

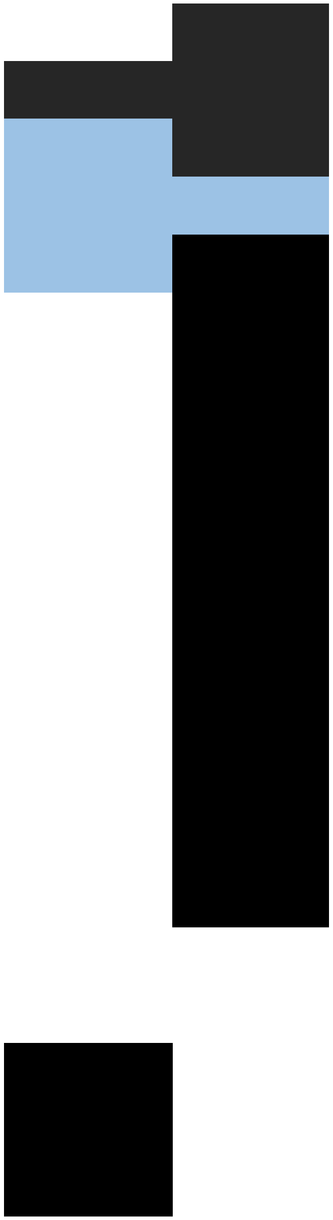

| | |
|---|---|
| M00309 Non-phosphorylative Entner-Doudoroff pathway, gluconate/galactonate => glycerate | **C** |
| M00854 Glycogen biosynthesis, glucose-1P => glycogen/starch | **C,P** |
| M00855 Glycogen degradation, glycogen => glucose-6P | **C,P** |
| M00565 Trehalose biosynthesis, D-glucose 1P => trehalose | **C,P** |
| M00549 Nucleotide sugar biosynthesis, glucose => UDP-glucose | **C,P** |
| M00631 D-Galacturonate degradation, D-galacturonate => pyruvate + D glyceraldehyde 3P | **C,P** |
| M00061 D-Glucuronate degradation, D-glucuronate => pyruvate + D-glyceraldehyde 3P | **C,P** |
| M00632 Galactose degradation, Leloir pathway, galactose => alpha-D-glucose-1P | **C,P** |
| M00552 D-galactonate degradation, De Ley-Doudoroff pathway, D-galactonate => glycerate-3P | **C,P** |
| M00554 Nucleotide sugar biosynthesis, galactose => UDP-galactose | **C,P** |
| M00012 Glyoxylate cycle | **C** |
| M00373 Ethylmalonyl pathway | **C** |
| M00013 Malonate semialdehyde pathway, propanoyl-CoA => acetyl-CoA | **C** |
| M00741 Propanoyl-CoA metabolism, propanoyl-CoA => succinyl-CoA | **C** |
| M00130 Inositol phosphate metabolism, PI=> PIP2 => Ins(1,4,5)P3 => Ins(1,3,4,5)P4 | **C,P** |
| M00132 Inositol phosphate metabolism, Ins(1,3,4)P3 => phytate | **C** |
| **Energy metabolism** | |
| **Carbon fixation** | |
| M00165 Reductive pentose phosphate cycle (Calvin cycle) | **C** |
| M00166 Reductive pentose phosphate cycle, ribulose-5P => glyceraldehyde-3P | **C,P** |
| M00167 Reductive pentose phosphate cycle, glyceraldehyde-3P => ribulose-5P | **C,P** |

| | |
|---|---|
| M00168 CAM (Crassulacean acid metabolism), dark | **C** |
| M00173 Reductive citrate cycle (Arnon-Buchanan cycle) | **C** |
| M00579 Phosphate acetyltransferase-acetate kinase pathway, acetyl-CoA => acetate | **C,P** |
| **Methane metabolism** | |
| M00345 Formaldehyde assimilation, ribulose monophosphate pathway | **C** |
| M00358 Coenzyme M biosynthesis | **C** |
| M00174 Methane oxidation, methanotroph, methane => formaldehyde | **C** |
| M00346 Formaldehyde assimilation, serine pathway | **C** |
| M00344 Formaldehyde assimilation, xylulose monophosphate pathway | **C,P** |
| M00378 F420 biosynthesis | **C** |
| **Nitrogen metabolism** | |
| M00531 Assimilatory nitrate reduction, nitrate => ammonia | **N** |
| M00175 Nitrogen fixation, nitrogen => ammonia | **N** |
| M00530 Dissimilatory nitrate reduction, nitrate => ammonia | **N** |
| M00529 Denitrification, nitrate => nitrogen | **N** |
| M00528 Nitrification, ammonia => nitrite | **N** |
| M00804 Complete nitrification, comammox, ammonia => nitrite => nitrate | **N** |
| **Sulfur metabolism** | |
| M00176 Assimilatory sulfate reduction, sulfate => H2S | S |
| M00595 Thiosulfate oxidation by SOX complex, thiosulfate => sulfate | **S** |
| M00596 Dissimilatory sulfate reduction, sulfate => H2S | **S** |

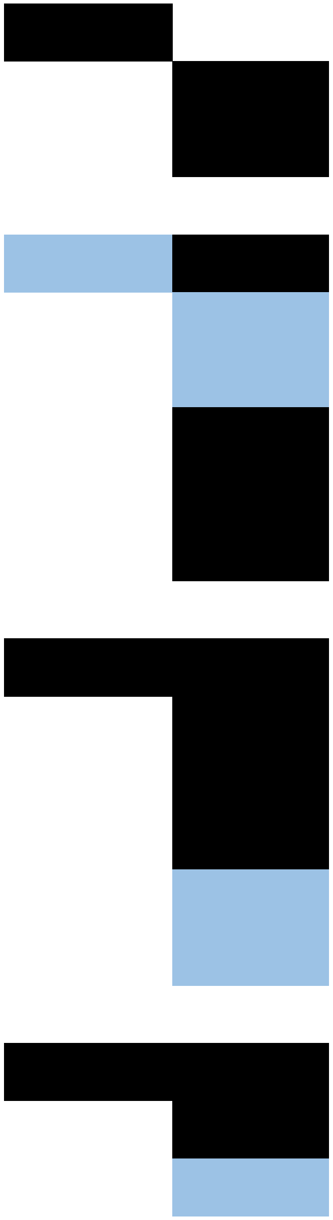

| | |
|---|---|
| **Photosynthesis** | |
| M00161 Photosystem II | C |
| M00163 Photosystem I | C |
| M00597 Anoxygenic photosystem II | **C** |
| **ATP synthesis (Structural complex)** | |
| M00144 NADH:quinone oxidoreductase, prokaryotes | |
| M00146 NADH dehydrogenase (ubiquinone) 1 alpha subcomplex | |
| M00145 NAD(P)H:quinone oxidoreductase, chloroplasts and cyanobacteria | |
| M00149 Succinate dehydrogenase, prokaryotes | |
| M00162 Cytochrome b6f complex | |
| M00154 Cytochrome c oxidase | |
| M00155 Cytochrome c oxidase, prokaryotes | |
| M00153 Cytochrome bd ubiquinol oxidase | |
| M00157 F-type ATPase, prokaryotes and chloroplasts | |
| M00150 Fumarate reductase, prokaryotes | |
| M00162 Cytochrome b6f complex | |
| M00151 Cytochrome bc1 complex respiratory unit | |
| M00152 Cytochrome bc1 complex | |
| M00154 Cytochrome c oxidase | |
| M00155 Cytochrome c oxidase, prokaryotes | |
| M00153 Cytochrome bd ubiquinol oxidase | |

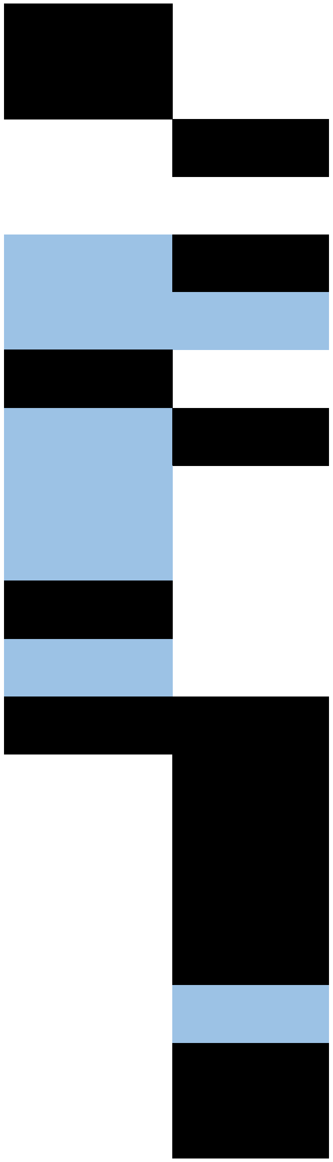

| | |
|---|---|
| M00417 Cytochrome o ubiquinol oxidase | |
| M00156 Cytochrome c oxidase, cbb3-type | |
| M00159 V-type ATPase, prokaryotes | |
| **Lipid metabolism** | |
| **Fatty acid metabolism** | |
| M00082 Fatty acid biosynthesis, initiation | **C** |
| M00083 Fatty acid biosynthesis, elongation | **C** |
| M00086 beta-Oxidation, acyl-CoA synthesis | **C** |
| M00087 beta-Oxidation | **C** |
| M00861 beta-Oxidation, peroxisome, VLCFA | **C** |
| **Sterol biosynthesis** | |
| M00862 beta-Oxidation, peroxisome, tri/dihydroxycholestanoyl-CoA => choloyl/chenodeoxycholoyl-CoA | **C** |
| M00107 Steroid hormone biosynthesis, cholesterol => prognenolone => progesterone | **C** |
| M00110 C19/C18-Steroid hormone biosynthesis, pregnenolone => androstenedione => estrone | **C** |
| **Lipid metabolism** | |
| M00098 Acylglycerol degradation | C |
| M00088 Ketone body biosynthesis, acetyl-CoA => acetoacetate/3-hydroxybutyrate/acetone | **C** |
| M00089 Triacylglycerol biosynthesis | **C** |
| M00090 Phosphatidylcholine (PC) biosynthesis, choline => PC | **C,P** |
| M00091 Phosphatidylcholine (PC) biosynthesis, PE => PC | **C,P** |

| | |
|---|---|
| M00092 Phosphatidylethanolamine (PE) biosynthesis, ethanolamine => PE | **C,P** |
| M00093 Phosphatidylethanolamine (PE) biosynthesis, PA => PS => PE | **C,P** |
| M00094 Ceramide biosynthesis | **C** |
| M00066 Lactosylceramide biosynthesis | **C** |
| M00099 Sphingosine biosynthesis | **C** |
| M00100 Sphingosine degradation | **C** |
| **Nucleotide metabolism** | |
| **Purine metabolism** | |
| M00048 Inosine monophosphate biosynthesis, PRPP + glutamine => IMP | **C,P** |
| M00049 Adenine ribonucleotide biosynthesis, IMP => ADP,ATP | **C,P** |
| M00050 Guanine ribonucleotide biosynthesis IMP => GDP,GTP | **C,P** |
| M00546 Purine degradation, xanthine => urea | **C,N** |
| **Pyrimidine metabolism** | |
| M00051 Uridine monophosphate biosynthesis, glutamine (+ PRPP) => UMP | **C,P** |
| M00052 Pyrimidine ribonucleotide biosynthesis, UMP => UDP/UTP,CDP/CTP | **C,P** |
| M00053 Pyrimidine deoxyribonuleotide biosynthesis, CDP/CTP => dCDP/dCTP,dTDP/dTTP | **C,P** |
| M00046 Pyrimidine degradation, uracil => beta-alanine, thymine => 3-aminoisobutanoate | **C,P** |
| **Amino acid metabolism** | |
| **Serine and threonine metabolism** | |
| M00018 Threonine biosynthesis, aspartate => homoserine => threonine | **C,N** |
| M00555 Betaine biosynthesis, choline => betaine | **C,N** |

| | |
|---|---|
| M00020 Serine biosynthesis, glycerate-3P => serine | **C,N** |
| M00033 Ectoine biosynthesis, aspartate => ectoine | **C,N** |
| **Cysteine and methionine metabolism** | |
| M00021 Cysteine biosynthesis, serine => cysteine | **C,N** |
| M00338 Cysteine biosynthesis, homocysteine + serine => cysteine | **C,N** |
| M00034 Methionine salvage pathway | **C,N** |
| M00035 Methionine degradation | **C,N** |
| M00017 Methionine biosynthesis, apartate => homoserine => methionine | **C,N** |
| M00368 Ethylene biosynthesis, methionine => ethylene | **C,N** |
| **Branched-chain amino acid metabolism** | |
| M00019 Valine/isoleucine biosynthesis, pyruvate => valine / 2-oxobutanoate => isoleucine | **C,N** |
| M00535 Isoleucine biosynthesis, pyruvate => 2-oxobutanoate | **C,N** |
| M00570 Isoleucine biosynthesis, threonine => 2-oxobutanoate => isoleucine | **C,N** |
| M00432 Leucine biosynthesis, 2-oxoisovalerate => 2-oxoisocaproate | **C,N** |
| M00036 Leucine degradation, leucine => acetoacetate + acetyl-CoA | **C,N** |
| **Lysine metabolism** | |
| M00016 Lysine biosynthesis, succinyl-DAP pathway, aspartate => lysine | **C,N** |
| M00526 Lysine biosynthesis, DAP dehydrogenase pathway, aspartate => lysine | **C,N** |
| M00527 Lysine biosynthesis, DAP aminotransferase pathway, aspartate => lysine | **C,N** |
| M00433 Lysine biosynthesis, 2-oxoglutarate => 2-oxoadipate | **C,N** |
| **Arginine and proline metabolism** | |

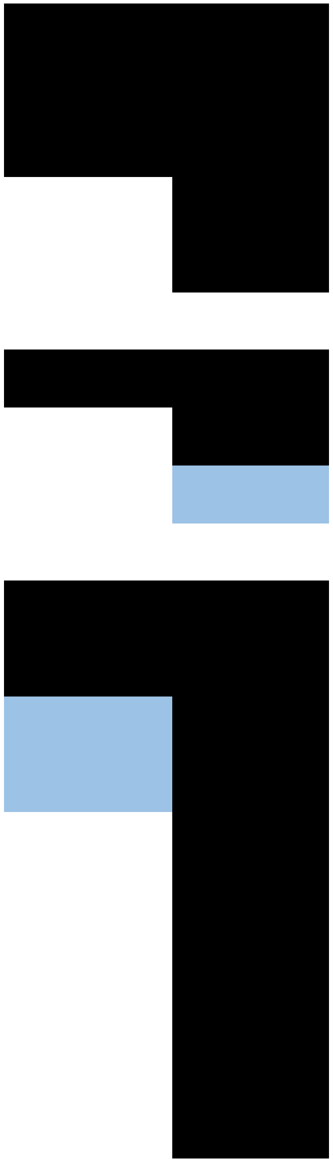

| | |
|---|---|
| M00028 Ornithine biosynthesis, glutamate => ornithine | **C,N** |
| M00844 Arginine biosynthesis, ornithine => arginine | **C,N** |
| M00015 Proline biosynthesis, glutamate => proline | **C,N** |
| M00845 Arginine biosynthesis, glutamate => acetylcitrulline => arginine | **C,N** |
| M00047 Creatine pathway | **C,N** |
| **Polyamine biosynthesis** | |
| M00133 Polyamine biosynthesis, arginine => agmatine => putrescine => spermidine | **C,N** |
| M00134 Polyamine biosynthesis, arginine => ornithine => putrescine | **C,N** |
| M00136 GABA biosynthesis, prokaryotes, putrescine => GABA | **C,N** |
| **Aromatic amino acid metabolism** | |
| M00022 Shikimate pathway, phosphoenolpyruvate + erythrose-4P => chorismate | **C,N** |
| M00023 Tryptophan biosynthesis, chorismate => tryptophan | **C,N** |
| M00024 Phenylalanine biosynthesis, chorismate => phenylalanine | **C,N** |
| M00040 Tyrosine biosynthesis, prephanate => pretyrosine => tyrosine | **C,N** |
| M00025 Tyrosine biosynthesis, chorismate => tyrosine | **C,N** |
| M00042 Catecholamine biosynthesis, tyrosine => dopamine => noradrenaline => adrenaline | **C,N** |
| M00044 Tyrosine degradation, tyrosine => homogentisate | **C,N** |
| M00533 Homoprotocatechuate degradation, homoprotocatechuate => 2-oxohept-3-enedioate | **C,N** |
| M00545 Trans-cinnamate degradation, trans-cinnamate => acetyl-CoA | **C,N** |
| M00038 Tryptophan metabolism, tryptophan => kynurenine => 2-aminomuconate | **C,N** |
| **Glycan metabolism** | |

| | |
|---|---|
| **Lipopolysaccharide metabolism** | |
| M00064 ADP-L-glycero-D-manno-heptose biosynthesis | **C** |
| M00072 N-glycosylation by oligosaccharyltransferase | **C** |
| M00073 N-glycan precursor trimming | **C** |
| M00056 O-glycan biosynthesis, mucin type core | **C** |
| M00070 Glycosphingolipid biosynthesis, lacto-series, LacCer => Lc4Cer | **C** |
| M00068 Glycosphingolipid biosynthesis, globo-series, LacCer => Gb4Cer | **C** |
| M00060 KDO2-lipid A biosynthesis, Raetz pathway, LpxL-LpxM type | **C** |
| M00866 KDO2-lipid A biosynthesis, Raetz pathway, non-LpxL-LpxM type | **C** |
| M00063 CMP-KDO biosynthesis | **C** |
| M00064 ADP-L-glycero-D-manno-heptose biosynthesis | **C** |
| **Metabolism of cofactors and vitamins** | |
| **Cofactor and vitamin metabolism** | **C,P** |
| M00127 Thiamine biosynthesis, AIR => thiamine-P/thiamine-2P | **C,P** |
| M00125 Riboflavin biosynthesis, GTP => riboflavin/FMN/FAD | **C,P** |
| M00115 NAD biosynthesis, aspartate => NAD | **C,N** |
| M00119 Pantothenate biosynthesis, valine/L-aspartate => pantothenate | **C** |
| M00120 Coenzyme A biosynthesis, pantothenate => CoA | **C** |
| M00123 Biotin biosynthesis, pimeloyl-ACP/CoA => biotin | **C** |
| M00842 Tetrahydrobiopterin biosynthesis, GTP => BH4 | **C** |
| M00843 L-threo-Tetrahydrobiopterin biosynthesis, GTP => L-threo-BH4 | **C** |

| | |
|---|---|
| M00140 C1-unit interconversion, prokaryotes | **C** |
| M00121 Heme biosynthesis, plants and bacteria, glutamate => heme | **C, Fe** |
| M00846 Siroheme biosynthesis, glutamate => siroheme | **C,N,Fe** |
| M00112 Tocopherol/tocotorienol biosynthesis | **C** |
| M00124 Pyridoxal biosynthesis, erythrose-4P => pyridoxal-5P | **C,P** |
| M00572 Pimeloyl-ACP biosynthesis, BioC-BioH pathway, malonyl-ACP => pimeloyl-ACP | **C,P** |
| M00573 Biotin biosynthesis, BioI pathway, long-chain-acyl-ACP => pimeloyl-ACP => biotin | **C,P** |
| M00577 Biotin biosynthesis, BioW pathway, pimelate => pimeloyl-CoA => biotin | **C** |
| M00126 Tetrahydrofolate biosynthesis, GTP => THF | **C** |
| M00841 Tetrahydrofolate biosynthesis, mediated by PTPS, GTP => THF | **C** |
| M00122 Cobalamin biosynthesis, cobinamide => cobalamin | **C,Co** |
| M00117 Ubiquinone biosynthesis, prokaryotes, chorismate => ubiquinone | **C** |
| M00116 Menaquinone biosynthesis, chorismate => menaquinol | **C** |
| **Biosynthesis of terpenoids and polyketides** | |
| **Terpenoid backbone biosynthesis** | |
| M00096 C5 isoprenoid biosynthesis, non-mevalonate pathway | **C** |
| M00364 C10-C20 isoprenoid biosynthesis, bacteria | **C** |
| M00365 C10-C20 isoprenoid biosynthesis, archaea | **C** |
| M00095 C5 isoprenoid biosynthesis, mevalonate pathway | **C** |
| **Xenobiotics biodegradation** | |
| **Aromatics degradation** | |

| | |
|---|---|
| M00538 Toluene degradation, toluene => benzoate | **C** |
| M00537 Xylene degradation, xylene => methylbenzoate | **C** |
| M00551 Benzoate degradation, benzoate => catechol / methylbenzoate => methylcatechol lete) | **C** |
| M00637 Anthranilate degradation, anthranilate => catechol | **C** |
| M00568 Catechol ortho-cleavage, catechol => 3-oxoadipate | **C** |
| M00569 Catechol meta-cleavage, catechol => acetyl-CoA / 4-methylcatechol => propanoyl-CoA | **C** |
| M00540 Benzoate degradation, cyclohexanecarboxylic acid =>pimeloyl-CoA | **C** |
| M00638 Salicylate degradation, salicylate => gentisate | **C** |
| M00623 Phthalate degradation, phthalate => protocatechuate | **C** |
| **Drug resistance** | |
| M00627 beta-Lactam resistance, Bla system | |
| M00745 Imipenem resistance, repression of porin OprD (13) | |
| M00651 Vancomycin resistance, D-Ala-D-Lac type | |
| M00726 Cationic antimicrobial peptide (CAMP) resistance, lysyl-phosphatidylglycerol (L-PG) synthase MprF | |
| M00744 Cationic antimicrobial peptide (CAMP) resistance, protease PgtE | |
| M00718 Multidrug resistance, efflux pump MexAB-OprM | |
| M00642 Multidrug resistance, efflux pump MexJK-OprM | |
| M00643 Multidrug resistance, efflux pump MexXY-OprM | |
| M00769 Multidrug resistance, efflux pump MexPQ-OpmE | |
| M00649 Multidrug resistance, efflux pump AdeABC | |

M00696 Multidrug resistance, efflux pump AcrEF-TolC

M00697 Multidrug resistance, efflux pump MdtEF-TolC

M00698 Multidrug resistance, efflux pump BpeEF-OprC

M00700 Multidrug resistance, efflux pump AbcA

M00714 Multidrug resistance, efflux pump QacA

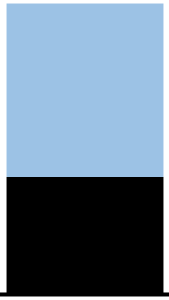

Supplement: Supplementary file 1 — AppendixS1: Supporting Information 1 [file LNO-65-S194-s001.pdf]
